# Ovatoxin-a and Palytoxin Accumulation in Seafood in Relation to *Ostreopsis* cf. *ovata* Blooms on the French Mediterranean Coast

**DOI:** 10.3390/md10020477

**Published:** 2012-02-17

**Authors:** Zouher Amzil, Manoella Sibat, Nicolas Chomerat, Hubert Grossel, Francoise Marco-Miralles, Rodolphe Lemee, Elisabeth Nezan, Veronique Sechet

**Affiliations:** 1 IFREMER, Phycotoxins Laboratory, BP 21105, F-44311 Nantes Cedex 3, France; Email: manoella.sibat@ifremer.fr (M.S.); veronique.sechet@ifremer.fr (V.S.); 2 IFREMER, LER-FBN, Marine Biological Station, BP 40537, F-29185 Concarneau Cedex, France; Email: nicolas.chomerat@ifremer.fr (N.C.); elisabeth.nezan@ifremer.fr (E.N.); 3 IFREMER, LER-PAC, La Seyne sur Mer, F-83507, Toulon, France; Email: hubert.grossel@ifremer.fr (H.G.); Francoise.Marco.Miralles@ifremer.fr (F.M.-M.); 4 Laboratory of Villefranche, Pierre et Marie Curie University, BP 28, F-06234, Villefranche-sur-Mer cedex, France; Email: rodolphe.lemee@obs-vlfr.fr; 5 CNRS, Marine Microbilal Ecology and Biogeochemistry, Pierre et Marie Curie-Paris 6 University, BP 28, F-06234, Villefranche-sur-Mer, France

**Keywords:** *Ostreopsis* cf. *ovata*, palytoxin, ovatoxin-a, LC-MS/MS, seafood

## Abstract

Dinoflagellates of the genus *Ostreopsis* are known to cause (often fatal) food poisoning in tropical coastal areas following the accumulation of palytoxin (PLTX) and/or its analogues (PLTX group) in crabs, sea urchins or fish. *Ostreopsis* spp. occurrence is presently increasing in the northern to north western Mediterranean Sea (Italy, Spain, Greece and France), probably in response to climate change. In France, *Ostreopsis.* cf. *ovata* has been associated with toxic events during summer 2006, at Morgiret, off the coast of Marseille, and a specific monitoring has been designed and implemented since 2007. Results from 2008 and 2009 showed that there is a real danger of human poisoning, as these demonstrated bioaccumulation of the PLTX group (PLTX and ovatoxin-a) in both filter-feeding bivalve molluscs (mussels) and herbivorous echinoderms (sea urchins). The total content accumulated in urchins reached 450 µg PLTX eq/kg total flesh (summer 2008). In mussels, the maximum was 230 µg eq PLTX/kg (summer 2009) compared with a maximum of 360 µg found in sea urchins during the same period at the same site. This publication brings together scientific knowledge obtained about the summer development of *Ostreopsis* spp. in France during 2007, 2008 and 2009.

## 1. Introduction

**Figure 1:**
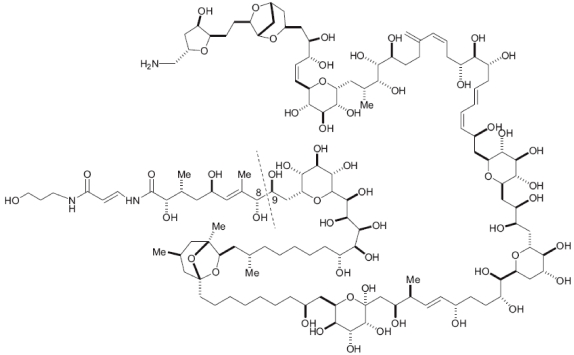
Palytoxin structure.

Palytoxin (PLTX) (Figure 1) was isolated for the first time from corals of the genus *Palythoa* [1]. It is a complex macromolecule whose chemical structure was elucidated in the 1980s [2,3,4]. Depending on the species from which it was isolated, the molecular weight and formula of PLTX can differ slightly, and some species may contain a mixture of different isomers [2]. The high toxicity of PLTX to mammals makes it one of the most toxic substances of marine origin presently known. In a recent review by Munday, lethal dose 50% (LD_50_) of PLTX to mice by intraperitoneal injection is between 0.31 and 1.5 µg/kg, depending on observation time and source of PLTX [5]. Its target is the ATPase Na^+^/K^+^ pump, a transmembrane enzyme that plays a role in maintaining the resting potential of nerve, muscle and heart cells [6].

Different analogues of PLTX, known as “ostreocines” were then identified in members of the dinoflagellate genus *Ostreopsis*. Ostreocine was the first major toxin identified in *Ostreopsis siamensis* [7,8]. Dinoflagellates belonging to genus *Ostreopsis* are benthic microalgae living on sandy or rocky substrates or on macrophytes in tropical, subtropical and recently temperate, coastal zones and are known to be the cause of food poisoning due to the accumulation of PLTX-group compounds in crabs, sea urchins or fish [9]. These neurotoxins cause a type of poisoning in humans called palytoxicosis, characterized by symptoms including: salivation, abdominal cramps, nausea, severe diarrhoea, muscle spasms and breathing difficulties, followed by death in the most severe cases [10,11,12]. Compounds structurally related to PLTX and acting the same way have been isolated following human poisoning resulting from the consumption of fish [13,14,15,16,17]. The involvement of the dinoflagellate *Ostreopsis* spp. was confirmed, both as a producer of the PLTX group and as a cause of palytoxicosis [18,19,20]. In 1998 in Madagascar, an intoxication resulting in the death of one person was reported following the consumption of contaminated sardines. This kind of poisoning, called clupeotoxicosis, has been linked to the presence of *O. siamensis* found in the digestive tract of the fish [14].

Over the last ten years, probably due to climate change, tropical species of the genus *Ostreopsis* have become more frequent in the north-western Mediterranean (Spain, Italy, Greece, Tunisia, Monaco and France) [21,22,23,24,25,26,27,28,29]. Among the dozen potentially toxic species, three are confirmed to produce PLTX-like compounds: *O. siamensis*, *O. mascarenensis* and *O. ovata*. The associated toxins are, respectively, ostreocine-D, mascarenotoxins-A and -B and ovatoxin-a [8,20,24].

In addition to the risk of accumulation of toxins from algae in the food chain [23], their proliferation at very shallow depth may have a direct health impact on users of the coast. Toxins can be found in water, in floating clumps and even in sea spray dispersed by the wind. Direct effects on humans can lead to skin irritations, respiratory infections and fever [22,24,30,31,32].

In the French Mediterranean, during the high temperatures of August 2006, a proliferation of *Ostreopsis* spp. was observed for the first time off Marseille. To acquire data on this new phenomenon in France, field and laboratory studies have been conducted since 2007 to identify the *Ostreopsis* species involved and to monitor the level of bioaccumulation of PLTX-group toxins in the food chain. After optimization of the process of chemical analysis, determination of toxin profiles was performed by liquid chromatography coupled with tandem mass spectrometry (LC-MS/MS). The present paper brings together the scientific knowledge thus obtained on the development of *Ostreopsis* spp. summer in France during the years 2007, 2008 and 2009.

## 2. Results and Discussion

### 2.1. Optimization of the PLTX Quantification Method by LC-MS/MS

#### 2.1.1. Selection of an Extraction Solvent for PLTX

At the time of the experiments, there was very little information on procedures for extraction of PLTX-group toxins from shellfish tissues available in the literature. In order to choose the solvent that would extract the maximum amount of toxin, different solvents were tested on homogenates of digestive glands of mussels spiked with PLTX (in the absence of certified reference materials): (i) acetone, which allows extraction of a wide range of substances and is used in the extraction of lipophilic toxins [33]; (ii) methanol/water (50/50), acidified with 0.2% acetic acid, which is used for extraction the PLTX from *Ostreopsis ovata* and *O. siamensis* [18]; (iii) ethanol/water (80/20) used by Onuma *et al*. [14] to extract PLTX from the viscera of fish; and (iv) three other solvents, using increasing proportions of methanol: 80, 90 and 100%.

The solvents were tested using a homogenate of 50 g digestive glands (DG) from uncontaminated mussels. Several test samples of 2 g of DG were spiked with a standard solution of PLTX of known concentration (10 µg PLTX/mL). For each extraction solvent, two tests were made on 2 g of spiked DG to test the effectiveness of the solvent extraction test. Apart from the type of solvent, all test samples underwent the same extraction protocol: 4 mL, 3 mL and 3 mL of extraction solvent tested, with centrifugation steps at 3000 g for 15 min in-between. Depending on the type of solvent tested, the supernatants were combined, and an aliquot filtered through 0.2 µm and analyzed by LC-MS/MS.

**Figure 2:**
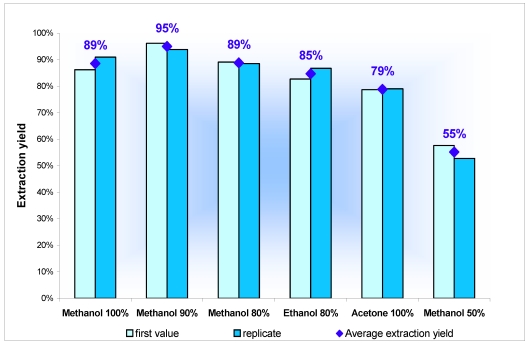
Recovery rate according to extraction solvent, for PLTX contained in spiked blue mussel digestive glands.

Mussel extracts, as well as the standard solution used for spiking, were analyzed in triplicate by LC-MS/MS to calculate the extraction yields. The experimental results obtained on 12 samples of mussel DG (2 tests per solvent) spiked with the PLTX are summarized in Figure 2. Only methanol/water (50/50) had a low extraction efficiency (55%) compared with the yields obtained using the other solvents (between 79 and 95%). The recovery rate results show that the methanol/water (90/10) is the most effective solvent, since it allowed the most PLTX (95%) to be extracted. 

#### 2.1.2. Specificity of the LC-MS/MS Analytical Procedure

**Figure 3:**
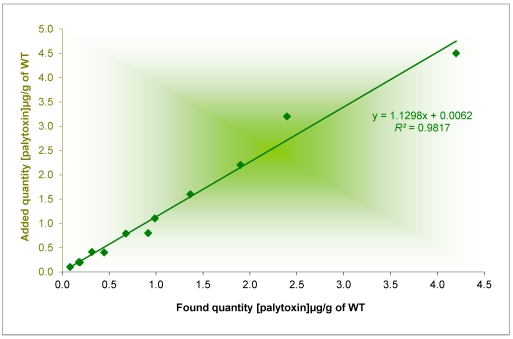
Recovery of different levels of PLTX in different tissues of marine invertebrates. The values of significance tests of the slope (t observed) of the intercept (t' observed) are below the critical Student value for α = 1%, p-2 = 10. The limits of the slope contained 1 and the limits of the intercept included 0.

The specificity of the procedure according to the tissue and the level of spiking was examined by testing it on different homogenates from healthy marine invertebrates: digestive glands of blue mussel (*Mytilus edulis*, Linnaeus, 1758 and *M. galloprovincialis*), digestive glands of wedge clam (*Donax cuneatus*, Linneaus, 1758), whole tissues of Manila clam (*Tapes philippinarum*, Adams and Reeve, 1850) and whole tissues of sea urchin (*Paracentrotus lividus*) from the Mediterranean coast. Each matrix was spiked with three different concentrations of PLTX. For toxins quantification in mussel and wedge clam, a calculated DG/whole tissue ratio was used to express all the results to the whole tissue from the amount found in DG. The recovery rate depends on the nature of the matrix, ranging from 75% to 115% (Table 1). The average recovery rate of PLTX varied depending source if the tissue: Manila clam (82%), wedge clam (90%), sea urchins (92%) and mussels (97%). To test whether the source tissue effect was significant or not, the relationship with recovery was plotted and a statistical study was conducted using the Student test (*t*) for the risk level α = 1% (Figure 3). These results show that the method of analysis of PLTX is specific. 

**Table 1:** Recovery of different levels of PLTX in different tissues of shellfish.

Seafood	Found Quantity (µg/g)	Added Quantity (µg/g)	Recovery
Manila clam	0.08	0.1	81%
	0.18	0.2	87%
	0.32	0.4	77%
Mussels	0.19	0.2	95%
	0.45	0.4	112%
	0.68	0.8	86%
Sea urchin	0.92	0.8	115%
	1.37	1.6	85%
	2.4	3.2	75%
Wedge clam	0.99	1.1	90
	1.90	22	86%
	4.20	4.5	93%

Our results on the optimization of the palytoxin quantification agree with those published by Ciminiello *et al*. in 2011, which were not known at the time of our experiments. According to their results, the extraction with MeOH/H_2_O 8/2 (9/1 not tested) provided the best results in terms of average recovery on mussels and sea urchins: 92% and 89% respectively [34]. This confirms our results. 

#### 2.1.3. Detection and Quantification Limits (LOD and LOQ)

The detection and quantification limits were determined for two matrices: whole tissues (2 g) of sea urchin and blue mussels spiked with a standard solution of PLTX of known concentration. The LOD and LOQ values did not include recovery of the extraction procedure. They were calculated using serial dilutions. The LOD (signal/noise, S/N = 3) and LOQ (S/N = 10) obtained were equal for both matrices: (i) 10 and 25 µg PLTX/kg whole tissue of sea urchins; (ii) 9 and 23 µg PLTX/kg whole tissue of mussels. 

### 2.2. First Report of the PLTX Group in Marine Organisms in the French Mediterranean

**Figure 4:**
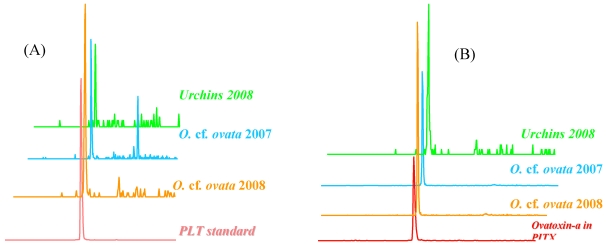
Examples of LC-MS/MS analyses for (**A**) PLTX (m/z 1340→327, characteristic ion); and (**B**) ovatoxin-a (m/z 1324→327, characteristic ion) in natural samples of *Ostreopsis* cf. *ovata* and sea urchins sampled in the summers of 2007 and 2008 in the Mediterranean.

The optimized LC-MS/MS chemical analysis method was applied to natural samples of microalgae and sea urchins collected during the summer development of *Ostreopsis* spp. The results obtained from samples collected in the Mediterranean from Villefranche-sur-Mer and from Morgiret (Iles de Frioul, off the coast of Marseille) during the summers of 2007 and 2008 revealed the presence of PLTX and ovatoxin-a (Figure 4): (i) in samples of natural *Ostreopsis* cf. *ovata* populations collected in 2007 and 2008; and (ii) for the first time in France, in the sea urchin samples collected in summer 2008 (July–August). For sea urchins, the tests for these toxins were performed separately on the gonads and digestive tract. In contrast to the gonads, where no toxin was detected, a significant amount of PLTX and, especially, ovatoxin-a was found in the digestive tract. The ovatoxin was quantified using the PLTX standard. The maximum levels found in terms of total PLTX equivalents (PLTX and ovatoxin-a) were approximately 175 µg and 450 µg eq PLTX/kg of total sea urchin flesh, in Iles de Frioul (August 2008) and Villefranche-sur-Mer (July 2008), respectively. The results were expressed taking into account the percentage of the whole body represented by the digestive tract relative to the total (including the gonads). 

In the *Ostreopsis* cf. *ovata* cells collected *in situ* at the two contaminated sites, ovatoxin-a was the compound present in the highest levels both in the cells fixed to macroalgae and in those suspended in the water column. Actually, in the literature this alga has been shown to produce, together with ovatoxin-a, other ovatoxins, such as ovatoxin-b, -c, -d, -e [35,36]. Therefore, toxin profile of *Ostreopsis* cf. *ovata* reported here is incomplete since such toxins were not known at the time of the experiments and thus they were not monitored.

The genus *Ostreopsis* had, in fact, already been found on the French Mediterranean coast; specifically, Max Taylor (in Congestri *et al*.) [37] inventoried *Ostreopsis* in the bay of Villefranche-sur-Mer in 1972. Furthermore, Vila *et al*. [21] found *Ostreopsis* spp. in the south-west of Corsica in the summer of 1998. During the high temperatures of August 2006, the first year that *Ostreopsis* spp. proliferations were detected in France, the observations showed an *Ostreopsis* bloom with concentrations up to 900,000 cells/L (sub-surface sample taken in an area where there were fixed fragments of macroalgae covered with mucilage), and 38,000 cells/L in open water at 0.2 m depth. This event led to a ban on swimming and the consumption of seafood in the affected area (Morgiret, Iles de Frioul, off the coast of Marseille) following signs of direct poisoning of several people who experienced symptoms of irritation in the mouth and throat [32]. Indeed, the toxins can be found in floating clumps of algae and even in sea spray droplets dispersed by the wind. Inhalation of contaminated spray was responsible for outbreaks of dramatic febrile respiratory syndromes and irritation of the skin and upper respiratory tract in 2004 in the area north of Barcelona (Spain) [24], in 2005 in Genoa (Italy) [38], and in 2009 in Algeria [39]. In Genoa, among the 200 people admitted to casualty, 20 were hospitalized [30,40].

### 2.3. Genetic Characterization of *Ostreopsis* Strains

**Figure 5:**
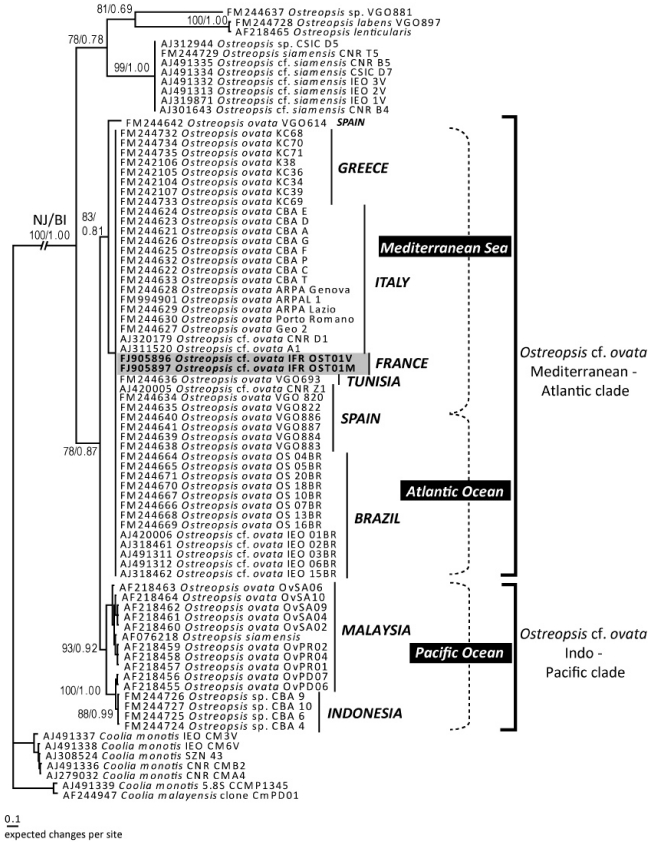
Phylogenetic tree (NJ tree) of the genus *Ostreopsis* based on the ITS region and 5.8S sequences. Numbers on the nodes represent bootstrap values (NJ) (1000 pseudoreplicates) and posterior probabilities (BI). The trees were rooted using *Coolia* sequences.

The PCR amplifications of 5.8S and ITS regions produced a single fragment of 341 bp for both cultures. The 5.8S fragment was 160 bp while ITS1 was 95 bp and ITS2 86 bp. The sequences obtained in this study have been deposited in GenBank under the accession numbers FJ905896 and FJ905897. In the phylogenetic tree (Figure 5), the two sequences cluster with other sequences of *Ostreopsis* cf. *ovata* from various areas of the Mediterranean Sea and Atlantic Ocean, from Greece to Brazil. These sequences are almost identical, but they differ from sequences of strains of *O*. cf. *ovata* collected in the Pacific Ocean which form a sister clade. Within the *Ostreopsis* lineage, *O. labens*, *O. lenticularis* and *O. siamensis* (including *O.* cf. *siamensis*) appear to be the most basal taxa.

### 2.4. Culture of *Ostreopsis* cf. *ovata* Strains

**Figure 6:**
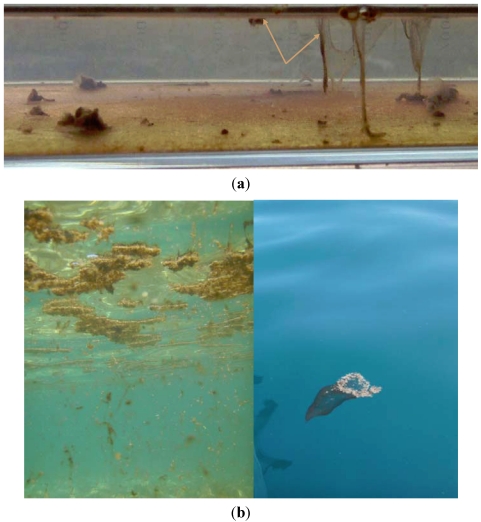
(**a**) Culture of *Ostreopsis* cf. *ovata* (IFR-OST-01V) isolated in 2008; (**b**) Detachment of *Ostreopsis* cells and filamentous agglomerates observed in August 2009 at Frioul islands (photographed by the CEEP).

The first step of the *in vitro* ecological studies conducted in the laboratory was to establish cultures of *Ostreopsis* spp. monoclonal *Ostreopsis* cf. *ovata* cultures were obtained from samples of seawater taken at Villefranche-sur-Mer (clone IFR-OST-01V) and Morgiret-Iles de Frioul (IFR-OST-01M) in summer 2008. Figure 6 shows a culture *Ostreopsis* cf. *ovata* kept in 250-mL bottles. When all of the substrate is colonized, the algae form mats that can detach from the bottom and become suspended in the water (arrows in Figure 6a). Based on field observations, it appears that the same phenomenon can occur in nature, namely a saturation of macroalgae and detachment of *Ostreopsis* cell clusters into the water column (see Figure 6b).

Chemical analysis by LC-MS/MS revealed a similar toxin profile both in cultivated strains and natural samples, with a majority proportion of ovatoxin-a (90%) and a small proportion of PLTX (10%). Ovatoxin-b, -c, -d, -e were not monitored.

### 2.5. *In Situ* Monitoring of Bioaccumulation of the PLTX Group in Filter-Feeding Shellfish during the Development *Ostreopsis* cf. *ovata* in Summer 2009

The presence of PLTX and ovatoxin-a in marine organisms does not necessarily reflect bioaccumulation by filtration, since sea urchins graze on the macroalgae that have *Ostreopsis* cells growing on them. To demonstrate the presence or absence of a bioaccumulation of toxins in bivalves filtering *Ostreopsis*, an experimental study was conducted *in situ* during summer 2009 by monitoring both: (i) the concentration *O.* cf. *ovata* in the environment; and (ii) changes in PLTX levels in the sea urchins present on site and in immersed mussels. Since there is no bivalve production in the monitored areas affected by *Ostreopsis*, net bags of mussels were immersed there at the end of June 2009 specifically for the experiment. Sea urchins were collected from area around where the mussel bags had been placed. The study was conducted at the site of Morgiret in Frioul and the site of Villefranche-sur-Mer, which is regularly affected by *Ostreopsis*. It should be pointed out that, in principle, the density of planktonic *Ostreopsis* will be more directly linked to the level of PLTX contamination in filter-feeding bivalves, such as mussels, and the density of benthic *Ostreopsis* will be more closely linked to contamination in sea urchins, as they graze the macroalgae. 

**Figure 7:**
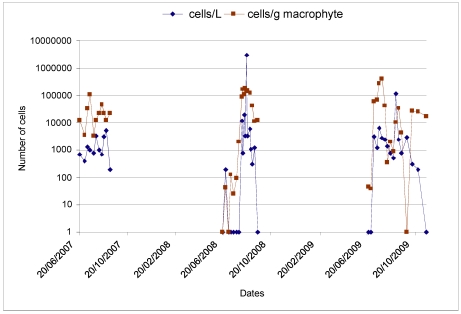
Comparison of *Ostreopsis* abundance (both epiphytical and in the water) during the summers 2007, 2008 and 2009 at Morgiret bay (Frioul islands, off the coast of Marseille).

For the site of Morgiret during the summer 2009, samples were taken every week from 24 June and then twice a month from mid-September. A comparison of the years 2007, 2008 and 2009 (Figure 7) highlights the role of the weather conditions: the successive windy episodes that marked 2007 summer (periods of mistral generating a cooling of water masses due to induced local upwelling; average temperature in July and August = 20.2 °C) corresponded to medium *Ostreopsis* abundance levels in the water and on the macroalgae. The summer 2008 showed very different environmental conditions from those of 2007, with more stable water bodies and less wind, causing less cooling (the average temperature for July and August was 21.3 °C, which is more than one degree warmer than 2007 and, therefore, non-negligible). In 2008, we observed a relatively stable high density of macroalgal *Ostreopsis*. 

In 2009, whereas the average temperature in July and August was superior to that of the 2008 (22.2 °C), *Ostreopsis* population levels were lower in the water and those on the macroalgae more irregular. There were very few *Ostreopsis* cells present in the first two macroalgal samples, taken at the end of June, and they were absent from the water column at this time. The next period, which lasted until the end of July, showed that levels of cells in the water remained relatively low (maximum 21 July with 6,400 cells/L), while the benthic *Ostreopsis* level rose and peaked on 28 July at about 400,000 cells/g wet macroalgae. In August relatively low values of *Ostreopsis* abundance were observed, both on the macroalgae and in the water, but the first week of September was marked by increases, both on the macroalgae (approximately 35,000 cells/g wet, 8 September) and in the water (by almost 120,000 cells/L, 1 September) making this result stand out from the rest series by its magnitude.

The end of the observation period, spanning from the end of September through October until mid-November, showed relatively low values of *Ostreopsis* spp. abundance in the water (there were, nonetheless, 3000 cells/L on 29 September), which then declined. But at the end of this series of observations (from the middle of October to mid-November), the concentrations of *Ostreopsis* populations epiphytically remained significant (17,000 cells/L on 17 November 2009).

The influence of environmental conditions on *Ostreopsis* cf. *ovata* proliferation has been reviewed by Pistocchi *et al*. [41]. They studied the relationship between growth and temperature on different strains isolated from sites located along the Adriatic (Ancona, Bari) and Tyrrhenien (Latina) coasts of Italy grown at temperature of 20 and 25 °C. The strain from Ancona displayed higher growth rates at the lowest temperature. Growth of the isolate from Latina did not show any difference between 20 and 25 °C while the strain from Bari had a better growth at 25 °C than at 20 °C. 

Chemical analysis by LC-MS/MS on toxicity of *Ostreopsis* cf. *ovata* from the two sites (Morgiret and Villefranche-sur-Mer) confirmed the reproducibility of the phenomenon, since the toxin profile of *O.* cf. *ovata* samples is the same as in previous years, as is the predominance of ovatoxin-a in comparison with PLTX.

### 2.6. Monitoring of Contamination and Detoxification of Mussels and Sea Urchins

**Figure 8:**
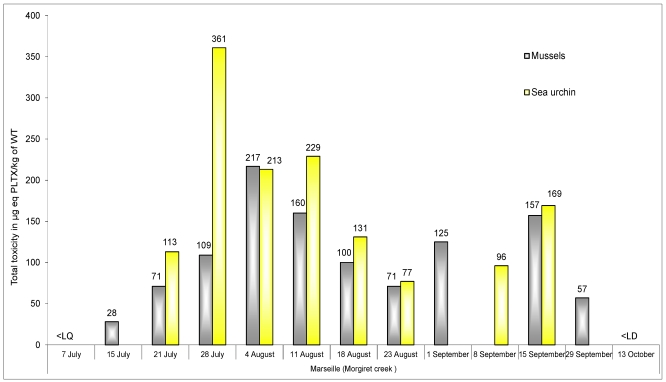
Monitoring of the bioaccumulation of PLTX-group toxins in mussels (immersed from 24 June) and sea urchins at Morgiret during the proliferations of *Ostreopsis* cf. *ovata* in summer 2009.

For monitoring bioaccumulation of PLTX-group compounds in seafood (sea urchins and mussels), the overall results indicate that PLTX and ovatoxin-a, if present, are concentrated only in the digestive tract of sea urchins and in the digestive glands of mussels, as these toxins were not detected in other tissues (the sea urchin gonad or the rest of the mussel flesh). Figure 8 shows the changing concentrations of these toxins found in mussels and sea urchins during the *Ostreopsis* cf. *ovata* algal blooms in the summer of 2009 on the Morgiret site.

For both seafood species studied, two phases of contamination were distinguished with two peaks of toxicity: in late July and mid September. This observation is consistent with the cell concentrations of *Ostreopsis* cf. *ovata* observed in the field (Figure 7). Indeed, until late July, the number of cells growing on the macroalgae was about 400,000 cells/g wet macroalgae, while in August relatively low values of *O.* cf. *ovata* were recorded both on the macroalgae and in the water. In contrast, the first week of September was marked by an increase of cells growing on the macroalgae (approximately 35,000 cells/g wet) and, especially, in the water (by almost 120,000 cells/L, 1 September).

According to the evolution of the accumulation of toxins (Figure 8), toxin levels found in sea urchins were generally higher than those found in mussels, with maxima of 360 µg and 217 µg eq. PLTX/kg total body, respectively. The end of the toxic episode appears to be at the end of September, since PLTX-like compounds were not detected in the digestive glands of mussels on 13 October (Figure 8).

Concerning the site of Villefranche-sur-Mer, the results confirm again an accumulation of palytoxin-like filtration in immersed mussels. The maximum accumulation of toxins occurred around mid-August, but the levels found are around half those measured in mussels immersed at the site of Morgiret (data not shown). Unlike the site Morgiret, monitoring *in situ* could not be completed following the disappearance of the mussel bags in the last week of August.

This contamination of bivalves by filtration is consistent with the sparse existing data in the literature on the bioaccumulation of PLTX in different shellfish species. Aligizaki *et al*. [23] reported the first episode of shellfish contamination by these toxins *in situ* in relation to the presence of *O.* cf. *ovata* species in Greek coastal waters in the 2004–2005 period. In this episode, the maximum concentrations of *Ostreopsis* spp. recorded were 40,000 cells/g fresh weight macroalgae and 3600 cells/L in the water column. Using the hemolytic tests, the maximum contamination level found was 97 µg PLTX/kg bivalve flesh. As the production areas were closed during this period, there were no reported cases of poisoning. However, the risk of human poisoning by the PLTX-like compounds through the consumption of contaminated seafood is a real one, and has already been reported by a number of authors [9,10,11,12,14].

During the blooms of *Ostreopsis* cf. *ovata* recorded in 2008 and 2009, concentrations of total PLTX (palytoxin + ovatoxin-a) exceeded the threshold value for the protection of public health established in late 2009 by the European Food Safety Authority (EFSA) [42]: 30 µg PLTX/kg shellfish flesh. Repeated bans on consumption of seafood at the sites where *Ostreopsis* blooms occurred were made several times over the 2008–2009 season. With such a threshold of health protection, seafood consumption should probably be systematically banned at sites contaminated by *Ostreopsis*. However, the mechanism by which palytoxin acts on human health after exposure to blooms of *Ostreopsis* or ingestion of contaminated shellfish remains unclear.

Knowing that sea urchin fishery is prohibited in the summer and that the edible part (gonads) does not contain the toxin, the risk seems limited. But it is imperative to consider the fact that these organisms are harvested in summer despite the ban and that some people eat the whole soft parts of sea urchins.

Since 2009, algal levels requiring a notice and an alert are, respectively, 30,000 cells/L of sea water and 100,000 cells/L, taking into account weather forecasts on risks of sea spray.

## 3. Conclusions

Since 2007, a monitoring experiment, involving environmental and epidemiological surveillance of the Mediterranean coast, has been operated by the Directorate General of Food and Health. This monitoring incorporates preventive risk management related to the presence of *Ostreopsis* populations in the environment with related research activities notably on the food toxicity risk (bioaccumulation of palytoxin in seafood). The results of this work show that many questions remain to be answered about *Ostreopsis*, in terms not only of health consequences, but on the prediction of blooms, and even monitoring arrangements. Improved knowledge about the ecology of *Ostreopsis* would help us to be better prepared for bloom situations.

To address these food risks and the occurrence of febrile respiratory syndromes associated with contaminated sea spray, it is essential to enhance and complement scientific knowledge on *Ostreopsis* and to perform more effective environmental monitoring on the Mediterranean coast that will allow a better prediction of the risk collective health phenomena like the one that occurred in Genoa. Concerning food safety, given the low threshold imposed by the EFSA, such work will also strengthen monitoring of the seafood contamination (sea urchins, mussels and fish), from commercial fishing and recreational fishing at bathing sites contaminated by *Ostreopsis*.

With regard to the specific toxins involved in this contamination, unlike PLTX, there is no standard for ovatoxin-a and its relative toxicity is not known, although it is the most predominant (90%) toxin in the various marine organisms harvested in areas affected by *O.* cf. *ovata* in the Mediterranean. Its toxin content is presently estimated using the PLTX standard. Therefore, future laboratory work will focus on the purification of ovatoxin-a in order to assess its activity, compared with PLTX in a panel of biological tests. These data will help in analyzing its health impact since, at the international level, there is very little data available to European and international bodies responsible for assessing the risk and establishing alert and health safety thresholds for *Ostreopsis* spp.

## 4. Materials and Methods

### 4.1. Materials

#### 4.1.1. Reagents and Reference Material

High performance liquid chromatography (HPLC) grade acetic acid, ethanol and acetone were purchased from Merck (Darmstadt, Germany), HPLC grade methanol and acetonitrile from J.T. Baker (Deventer, Netherlands). Water was deionised and purified to 18.2 MΩ quality through a MilliQ water purification system (Purelab Elga, UK). 

Reference material: A standard solution of palytoxin was purchased from Wako chemicals GmbH (Neuss, Germany). 

#### 4.1.2. Seafood Samples

Sea urchins (*Paracentrotus lividus*, Lamarck 1816) and bivalve molluscs were collected from Mediterranean areas contaminated with *Ostreopsis* spp. during the summers of 2007, 2008 and 2009. Bags of test mussels (*Mytilus galloprovincialis*, Lamarck 1819) were immersed in the field when required.

#### 4.1.3. Microalgae Samples

During the summer development of *Ostreopsis* spp., field samples were taken both from the water column (suspension cells) and from the macroalgae (epiphyticcells). Macroalgae were agitated in a plastic bag for about 1 min to loosen the fixed cells, followed by filtration through 500 µm to remove the macroalgae. The filtrates containing *Ostreopsis* cells and the samples of cells suspended in the water column were examined under the microscope and tested for the presence of PLTX-group compounds by LC-MS/MS. The strains isolated and cultured were also identified by genetic tests.

### 4.2. Methods

#### 4.2.1. Cultures of *Ostreopsis*

Two strains of *Ostreopsis* were isolated from the water samples collected at the two sites (strain IFR-OST-0.1M from Morgiret and strain IFR-OST-01V from Villefranche-sur-Mer) using a capillary pipette. After an initial growth in microplates, the cells were cultured in flask at 22 °C under a 16:8 h L:D cycle. Cultures were established in filtered natural seawater, at salinity of 35, adding nutrients at L1 concentration [43]. 

#### 4.2.2. Taxonomic Identification by Molecular Analysis

##### 4.2.2.1. DNA Amplification and Sequencing

Approximately 15 mL of exponentially growing cultures IFR-OST01V and IFR-OST01M were harvested by centrifugation (5000 rpm, 10 min). DNA of pelleted cells was extracted using CTAB (*N*-cetyl-*N*,*N*,*N*-trimethylammoniumbromide) method [44]. The 5.8S rDNA and ITS regions (ITS1 and ITS2) were amplified by using oligonuclotide primers ITS-FW (5'-GTAGGTGAACCTGCGGAAGG-3'), and ITS-RV (5'-TCCTCTTGCTTGATCTGAGATCCGG-3'). Genomic DNA was amplified in 25 µL PCR reaction containing 1 µL of extracted DNA, 6.5 µL of ultrapure water, 2.5 µL of each primer (10 µM) and 12.5 µL of PCR Master Mix 1× (Promega, Madison, WI, USA) which includes Taq polymerase, dNTPs, MgCl_2_ and reaction buffer. The polymerase chain reactions were performed in a Mastercycler Personal (Eppendorf, Hamburg, Germany) as follows: one initial denaturating step at 94 °C for 2 min, followed by 45 cycles each consisting of 94 °C for 30 s, 54 °C for 30 s, and 72 °C for 4 min, and a final elongation of 72 °C for 5 min. The PCR products were visualized on a 1% (w/v) agarose gel, excised, and purified with the Wizard SV Gel and PCR Clean-up system (Promega) according to the manufacturer’s recommendations. Then, they were sequenced directly using the ABI PRISM BigDye Terminator Cycle Sequencing Kit (Applied Biosystems, Carlsbad, CA, USA). Sequencing products were purified by exclusion chromatography using the Dye Terminator Removal Kit (Thermo Scientific) and the sequences were determined using an automated 3130 genetic analyser (Applied Biosystems).

##### 4.2.2.2. Sequences Alignment and Phylogenetic Analysis

The two sequences obtained were aligned with 71 sequences of *Ostreopsis* species and 7 sequences of *Coolia* species retrieved in Genbank, using MUSCLE [45]. The alignment was refined by eye using with BioEdit [46,47].

Evolutionary models were examined with jModeltest [48]. Bayesian Inference (BI) analysis was run using Mr Bayes [49,50]. Initial Bayesian analyses were run with a GTR model (nst = 6) with rates set to invgamma and nucleotide frequencies set to equal. Each analysis was performed using four Markov chains (MCMC), with one million cycles for each chain. Trees were saved to a file every 100 cycles and the first 2000 trees were discarded. Therefore, a majority-rule consensus tree was created from the remaining 8000 trees in order to examine the posterior probabilities (pp) of each clade. Neighbor-joining (NJ) analysis was performed using MEGA5 [51], with Maximum Composite Likelihood method. Bootstrap analysis (1000 pseudoreplicates) was used to assess the relative robustness of branches [52].

#### 4.2.3. Extraction Procedure for Palytoxin Group Toxins

##### 4.2.3.1. Seafood

This is the protocol chosen after optimization of the extraction procedure using spiking experiments with palytoxin standard solution (see section 2.1.). The extraction was performed on 2 g of homogenates of whole tissue of shellfish (sea urchin, mussels…). The extraction was carried out with 4 mL, 3 mL and 3 mL of 90% MeOH successively, each followed by centrifugation at 3000 g for 15 min, after which the supernatants were combined. An aliquot (400 μL) was filtered through a Whatman 0.2 µm vectaspin filter and 5 μL of the filtrate injected into the LC-MS/MS analysis system.

##### 4.2.3.2. *Ostreopsis* Samples

Samples of 10 mL of filtrate containing *Ostreopsis* cells (obtained from macroalgae), 10 mL of cells suspended in water column or 10 mL of *Ostreopsis* spp. cultured in the laboratory were used for the extraction of toxins according to the following procedure: (i) centrifugation at 3000 g at 4 °C for 15 min; (ii) recovery of the cell pellet in 1 mL of methanol/water (90/10); (iii) sonication with an ultrasonic probe twice or 40 min. Once all the cells were disrupted, the treated sample was centrifuged at 3000 g at 4 °C for 15 min. The supernatant was filtered through 0.2 µm and the toxin profile determined by LC-MS/MS.

#### 4.2.4. Liquid Chromatography-Multiple Tandem Mass Spectrometry Analysis (LC-MS/MS)

Liquid chromatography coupled with tandem mass spectrometry (LC-MS/MS) analysis was performed with aqueous methanol 90% extracts. An aliquot (300 µL) was filtered through a Whatman 0.2 µm vectaspin filter, and 5 µL of the filtrate were injected for analysis by LC-MS/MS. 

PLTX analyses were carried out using an LC system (HP 1200, Agilent) coupled to a hybrid triple quadrupole/ion trap mass spectrometer (API-4000 Qtrap, PE/SCIEX) equipped with a turbo spray^®^interface, according to a modified Ciminiello method [38]. A 5 µm C18 Gemini column (150 × 2.0 mm, Phenomenex) was employed at 20 °C and eluted at 200 µL/min. Eluent A was water and eluent B was 95% acetonitrile/water; both eluents contained 2 mM ammonium formiate and 50 mM formic acid. The gradient in B was increased 20 to 100% over 10 min and was held for 4 min before to lowering it to the initial conditions. The instrument control, data processing and analyses were conducted using Analyst software. 

Mass spectrometry detection was operated in positive mode and optimized from a PLTX standard solution using Multiple Reaction Monitoring (MRM). The MRM experiments were established using the following source settings: curtain gas set at 30, ion spray at 5500 V, a turbogas temperature of 450 °C, gas 1 and 2 set at 50 (arbitrary units) and an entrance potential of 10 V. The collision energy was applied at 45 eV for the bi-charged ions [M + 2H]^2+^ and [M + 2H − H_2_O]^2+^ and at 33 eV for the tri-charged ion [M + 3H]^3+^ to give the characteristic product ion at 327. The following transitions: *m/z* 1340 [M + 2H]^2+^→327 [M + H − B moiety − H_2_O]^+^ (declustering potential (DP) = 26 V, cell exit potential (CXP) = 18 V) and *m/z* 1332 [M + 2H − H_2_O]^2+^→327 [M + H − B moiety − H_2_O]^+^ (DP = 26 V, CXP = 18 V) for PLTX, *m/z* 1324 [M + 2H]^2+^→327 [M + H − B moiety − H_2_O]^+^ (DP = 26V, CXP = 18 V) and *m/z* 1315 [M + 2H − H_2_O]^2+^→327 [M + H − B moiety − H_2_O]^+^ (DP = 26 V, CXP = 18 V) for ovatoxin-a, were monitored with a dwell time of 250 ms for each transition. 
